# Automated radiosynthesis of two ^18^F-labeled tracers containing 3-fluoro-2-hydroxypropyl moiety, [^18^F]FMISO and [^18^F]PM-PBB3, via [^18^F]epifluorohydrin

**DOI:** 10.1186/s41181-021-00138-9

**Published:** 2021-07-10

**Authors:** Takayuki Ohkubo, Yusuke Kurihara, Masanao Ogawa, Nobuki Nengaki, Masayuki Fujinaga, Wakana Mori, Katsushi Kumata, Masayuki Hanyu, Kenji Furutsuka, Hiroki Hashimoto, Kazunori Kawamura, Ming-Rong Zhang

**Affiliations:** 1 Department of Advanced Nuclear Medicine Sciences, Institute for Quantum Medical Science, National Institutes for Quantum and Radiological Science and Technology, 263-8555 Chiba, Japan; 2 SHI Accelerator Service Ltd, 141-0032 Tokyo, Japan

**Keywords:** ^18^F, [^18^F]Epifluorohydrin, [^18^F]FMISO, [^18^F]PM-PBB3, Positron emission tomography (PET)

## Abstract

**Background:**

[^18^F]Fluoromisonidazole ([^18^F]FMISO) and 1-[^18^F]fluoro-3-((2-((1*E*,3*E*)-4-(6-(methylamino)pyridine-3-yl)buta-1,3-dien-1-yl)benzo[d]thiazol-6-yl)oxy)propan-2-ol ([^18^F]PM-PBB3 or [^18^F]APN-1607) are clinically used radiotracers for imaging hypoxia and tau pathology, respectively. Both radiotracers were produced by direct ^18^F-fluorination using the corresponding tosylate precursors 1 or 2 and [^18^F]F^−^, followed by the removal of protecting groups. In this study, we synthesized [^18^F]FMISO and [^18^F]PM-PBB3 by ^18^F-fluoroalkylation using [^18^F]epifluorohydrin ([^18^F]5) for clinical applications.

**Results:**

First, [^18^F]5 was synthesized by the reaction of 1,2-epoxypropyl tosylate (8) with [^18^F]F^−^ and was purified by distillation. Subsequently, [^18^F]5 was reacted with 2-nitroimidazole (6) or PBB3 (7) as a precursor for ^18^F-labeling, and each reaction mixture was purified by preparative high-performance liquid chromatography and formulated to obtain the [^18^F]FMISO or [^18^F]PM-PBB3 injection. All synthetic sequences were performed using an automated ^18^F-labeling synthesizer. The obtained [^18^F]FMISO showed sufficient radioactivity (0.83 ± 0.20 GBq at the end of synthesis (EOS); *n* = 8) with appropriate radiochemical yield based on [^18^F]F^−^ (26 ± 7.5 % at EOS, decay-corrected; *n* = 8). The obtained [^18^F]PM-PBB3 also showed sufficient radioactivity (0.79 ± 0.10 GBq at EOS; *n* = 11) with appropriate radiochemical yield based on [^18^F]F^−^ (16 ± 3.2 % at EOS, decay-corrected; *n* = 11).

**Conclusions:**

Both [^18^F]FMISO and [^18^F]PM-PBB3 injections were successfully synthesized with sufficient radioactivity by ^18^F-fluoroalkylation using [^18^F]**5**.

**Supplementary Information:**

The online version contains supplementary material available at 10.1186/s41181-021-00138-9.

## Background

Fluorine-18 (T_1/2_ = 109.8 min) is indispensable for the development of positron emission tomography (PET) tracers because its decay characteristic is better than that of carbon-11 (T_1/2_ = 20.1 min). The direct ^18^F-fluorination using a tosylate or triflate precursor and [^18^F]F^−^ is a widely used method for the introduction of fluorine-18 into target molecules to afford a large number of ^18^F-labeled PET tracers (Cole et al. 2014; Deng et al. 2019; Miller et al. 2008). In addition, ^18^F-fluoroalkylation is also a useful tool for inserting fluorine-18 into target molecules containing nucleophilic hydroxyl and amino functional groups (Zhang and Suzuki 2007). ^18^F-Fluoroalkylation has some advantages over direct ^18^F-fluorination. For example, ^18^F-fluoroalkylation applies more accessible and available phenols, carboxylic acids, amines, and amides as precursors for ^18^F-labeling (Iwata et al. 2002; Wilson et al. 1995; Zhang and Suzuki 2007). We have synthesized ^18^F-fluoroalkyl agents, such as [^18^F]fluoro-methyl, ethyl, and propyl bromide ([^18^F]F(CH_2_)_n_Br, *n* = 1–3) (Yanamoto et al. 2009; Yui et al. 2010; Zhang et al. 2002, 2003, 2004; Zhang and Suzuki 2007), deuterium-substituted [^18^F]fluoromethyl bromide ([^18^F]FCD_2_Br), and its triflate ([^18^F]FCD_2_OTf) using an automated ^18^F-labeling synthesizer (Arakawa et al. 2008; Mori et al. 2019). Using these ^18^F-fluoroalkyl agents, we synthesized dozens of ^18^F-fluoroalkylated tracers starting from the precursors of phenols, carboxylic acids, amines, and amides for PET imaging of receptors, enzymes, and transporters in the brain (Zhang and Suzuki 2007). Among these PET tracers, [^18^F]FEDAA1106 (Fujimura et al. 2006), [^18^F]FE-SPARQ (Haneda et al. 2007), [^18^F]FMeNER-*d*_2_ (Arakawa et al. 2008), [^18^F]FEPE2I (Sasaki et al. 2012), and [^18^F]FEDAC (Chung et al. 2018; Xie et al. 2012) have been synthesized for clinical applications in our PET center.

**Fig. 1:**
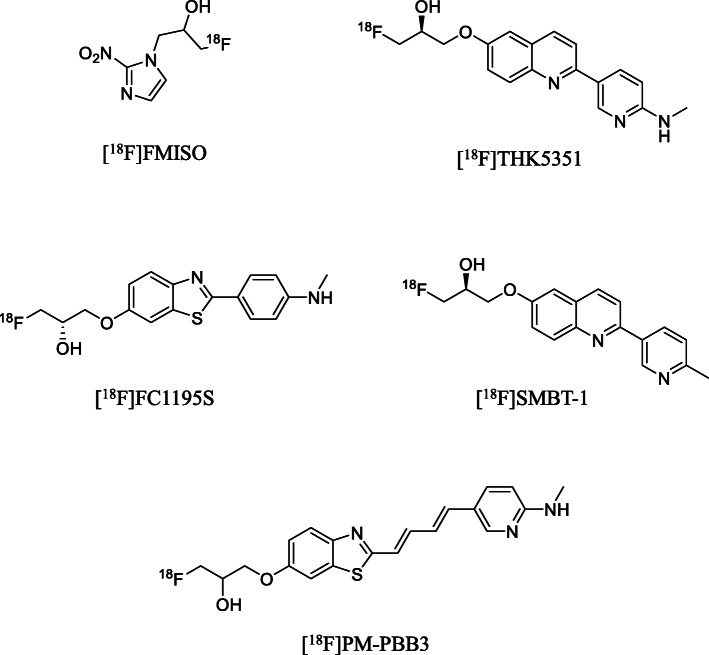
Chemical structures of PET tracers containing (3-[^18^F]fluoro-2-hydroxy)propyl ([^18^F]FHP) moiety used in clinical applications. [^18^F]FMISO (Bruehlmeier et al. 2004; Eschmann et al. 2005); [^18^F]THK-5351 (Harada et al. 2016; Tago et al. 2016); [^18^F]FC1195S (Byun et al. 2017; Lee et al. 2016; Yang et al. 2016); [^18^F]SMBT-1 (Harada et al. 2021); [^18^F]PM-PBB3 (Tagai et al. 2021)

The ^18^F-3-fluoro-2-hydroxypropyl (^18^F-FHP) moiety was used instead of the aforementioned conventional ^18^F-fluoroalkyl moieties. Many PET tracers containing the ^18^F-FHP moiety have been developed, some of which have been used in clinical studies, such as [^18^F]FMISO (Bruehlmeier et al. 2004; Eschmann et al. 2005), [^18^F]THK-5351 (Harada et al. 2016; Tago et al. 2016), [^18^F]FC1195S (Byun et al. 2017; Lee et al. 2016; Yang et al. 2016), [^18^F]SMBT-1 (Harada et al. 2021), and [^18^F]PM-PBB3 (Tagai et al. 2021; Kawamura et al. 2021) (Fig. 1). Among these PET tracers, [^18^F]THK-5351 (Fig. 1), which contains the FHP moiety, showed improved *in vivo* metabolic stability compared with its fluoropropyl analog (Tago et al. 2016).

To date, many ^18^F-labeled tracers containing ^18^F-fluoroalkyl moieties have been developed. To synthesize these PET tracers, direct ^18^F-fluorination of the corresponding tosylate or triflate precursor with [^18^F]F^−^ is a conventional method. Among these, [^18^F]FMISO as a PET imaging agent for tumor hypoxia (Oh et al. 2005; Tang et al. 2005), and [^18^F]PM-PBB3 as a PET imaging agent for tau pathology (Kawamura et al. 2021) have been prepared by direct ^18^F-fluorination using tosylate precursors and [^18^F]F^−^, followed by the removal of the protecting group. The direct ^18^F-fluorination was achieved within the same reaction vessel using an automated synthesizer. Moreover, the one-step radiolabeling technique for ^18^F-labeled tracers could be readily transferred to other PET centers for multisite studies using the same study protocol (Kawamura et al. 2016, 2021; Mori et al. 2017). In fact, automated radiosynthesis of [^18^F]PM-PBB3 by direct ^18^F-fluorination has been transferred to a dozen PET centers in Japan, China, Taiwan, and the USA (Hsu et al. 2020; Su et al. 2020; Weng et al. 2020). As for the limitation of these direct ^18^F-fluorination, it is noted that tosylated precursors should be synthesized in at least two steps. [^18^F]FMISO was synthesized using [^18^F]epfluorohydrin ([^18^F]5), as described previously (Grierson et al. 1989; Kämäräinen et al. 2004; McCarthy et al. 1993). In those papers, fully automated radiosynthesis procedures of [^18^F]FMISO via [^18^F]5 using an ^18^F-labeling synthesizer have not been reported.

**Fig. 2:**
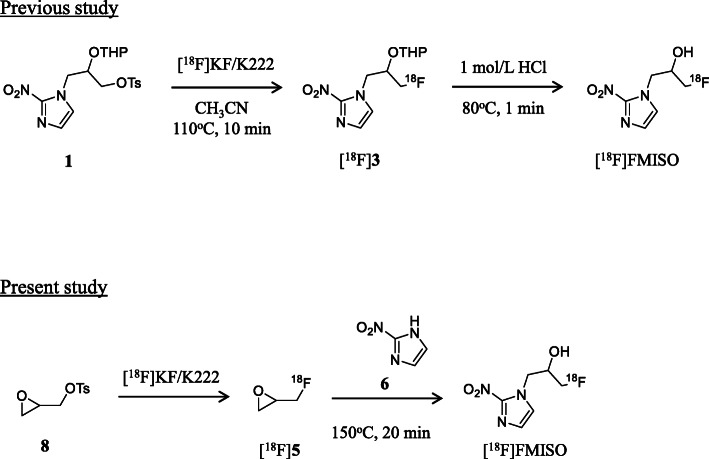
Radiosynthesis of [^18^F]FMISO by ^18^F-fluorination using [^18^F]F^−^ and ^18^F-fluoroalkylation using [^18^F]epifluorohydrin ([^18^F]5). In this study, after the [^18^F]F^−^ solution was dried, a solution of 8 (3.5 mg) in *o*-dichlorobenzene (0.15 mL) was added to a reaction vial containing dry [^18^F]F^−^. The resulting [^18^F]5 was distilled under an atmosphere of nitrogen, and transferred to another reaction vial containing 6 (2 mg) and 1 mol/L NaOH (18 µL) in anhydrous DMF (0.25 mL) maintained at -15 °C. After 2 min of trapping of [^18^F]5, the reaction mixture was heated at 150 °C for 20 min to obtain [^18^F]FMISO

**Fig. 3:**
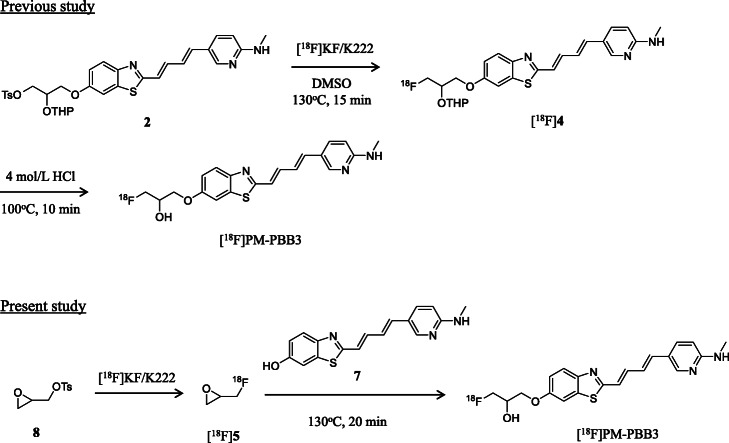
Radiosynthesis of [^18^F]PM-PBB3 by ^18^F-fluorination with [^18^F]F^−^ and ^18^F-fluoroalkylation using [^18^F]epifluorohydrin ([^18^F]5). In this study, after the [^18^F]F^−^ solution was dried, a solution of 8 (3.5 mg) in *o*-dichlorobenzene (0.15 mL) was added to the reaction vial containing the dry [^18^F]F^−^. The resulting [^18^F]5 was distilled from the reaction vial and was transferred to another reaction vial containing 7 (1 mg) and 1 mol/L NaOH (3.5 µL) in anhydrous DMF (0.25 mL) maintained at -15 °C. After 2 min of trapping of [^18^F]5, the reaction mixture was heated at 130 °C for 20 min to obtain [^18^F]PM-PBB3

In this study, to determine an effective synthetic route for [^18^F]FMISO and [^18^F]PM-PBB3 with sufficient radioactivity and high quality for clinical applications, we synthesized the two PET tracers using [^18^F]5 as an ^18^F-labeling agent by the reaction of easily accessible 2-nitroimidazole (6, Fig. 2) or PBB3 (a phenol precursor; 7, Fig. 3) using an ^18^F-labeling synthesizer equipped with a fully automated system. Furthermore, we compared the synthetic results of ^18^F-fluoroalkylation using [^18^F]5 and ^18^F-fluorination using [^18^F]F^−^ to evaluate their relative merits.

## Methods

### General

1* H*-1-(3-Fluoro-2-hydroxypropyl)-2-nitroimidazole (FMISO, Fig. 1) and 2-nitroimidazole (6, Fig. 2) were purchased from ABX (Radeberg, Germany). 1-Fluoro-3-((2-((1*E*,3*E*)-4-(6-(methylamino)pyridine-3-yl)buta-1,3-dien-1-yl)benzo[*d*]thiazol-6-yl)oxy)propan-2-ol (PM-PBB3, Fig. 1), and 2-((1*E*,3*E*)-4-(6-(methylamino)pyridin-3-yl)buta-1,3-dienyl)benzo[*d*]thiazol-6-ol (PBB3; **7**, Fig. 3) (Hashimoto et al. 2014; Maruyama et al. 2013) were provided by Shanghai ChemPartner (Shanghai, China). All chemical reagents and organic solvents were purchased from Sigma-Aldrich (St. Louis, MO, USA), Fujifilm Wako Pure Chemical Co. (Osaka, Japan), and Nacalai Tesque (Kyoto, Japan), and were used without any further purification. Fluorine-18 was produced by the ^18^O(p, n)^18^F nuclear reaction using a CYPRIS HM-18 cyclotron (Sumitomo Heavy Industry, Tokyo, Japan). A dose calibrator (IGC-3R Curiemeter; Aloka, Tokyo, Japan) was used for all radioactivity measurements, unless otherwise stated. An automated multi-purpose synthesizer developed in-house was used for all the radiosynthetic runs in this study (Supplemental information: Fig. S1, Fukumura et al. 2007). Preparative high-performance liquid chromatography (HPLC) was performed using a JASCO HPLC system (PU-2080 pump and UV-2075 detector; JASCO, Tokyo, Japan) equipped with a radioactivity detector (Ohyo Koken Kogyo, Tokyo, Japan). All radiochemical yields were decay-corrected to the end of synthesis. Fluorine-18, as [^18^F]F^−^, was produced as described previously (Fujinaga et al. 2018).

### Automated radiosynthesis of [^18^F]FMISO using [^18^F]5

After the [^18^F]F^−^ solution (5.2 ± 0.20 GBq, *n* = 8) was dried, a solution of epoxypropyl tosylate (**8**, 3.5 mg) in *o*-dichlorobenzene (0.15 mL) was added to the reaction vial containing dry [^18^F]F^−^ at 130 °C. The resulting [^18^F]**5** was distilled from the reaction vial under N_2_ flow at 30 mL/min and was transferred to another reaction vial containing precursor **6** (2 mg) and 1 mol/L sodium hydroxide solution (NaOH, 18 µL) in anhydrous *N*,*N*-dimethylformamide (DMF, 0.25 mL) maintained at − 15 °C. After 2 min of trapping of [^18^F]**5**, the reaction mixture was heated at 150 °C for 20 min, and then was diluted with the preparative HPLC eluent (0.5 mL). The solution mixture was transferred to the injector for preparative HPLC, as described in the general section. The HPLC conditions were as follows: XBridge C_18_ column (5 μm, 10 mm i.d. × 250 mm length; Waters), a mixture of ethanol and water (2:98, vol./vol.) as the mobile phase, 5.0 mL/min flow rate, and UV detection at 325 nm. The retention time of [^18^F]FMISO was approximately 12 min. The HPLC fraction of [^18^F]FMISO was collected in a flask containing polysorbate 80 (75 µL) in ethanol (0.3 mL), and ascorbic acid for injection (25 mg/0.1 mL water) was added before radiosynthesis. The solution was subsequently evaporated to dryness, and the residue was dissolved in physiological saline (3–10 mL). The resulting solution was passed through a Millex-GV filter (Millipore) to obtain [^18^F]FMISO as an injectable solution.

The radiochemical purity of [^18^F]FMISO was determined using analytical HPLC under the following conditions: XBridge C_18_ column (5 μm, 4.6 mm i.d. × 150 mm length; Waters), a mixture of 90 % acetonitrile solution and 50 mM ammonium phosphate buffer (pH 9.3) (7:93, vol./vol.) as the mobile phase, 1.0 mL/min flow rate, and UV detection at 325 nm. The retention time of [^18^F]FMISO was 5.6 min. The identity of [^18^F]FMISO was confirmed by co-injecting it with authentic unlabeled FMISO. The molar activity of [^18^F]FMISO was measured using the same analytical HPLC system. The mass (µmol) of FMISO with a known radioactivity (GBq) was determined using analytical HPLC by comparing the UV absorbance at 325 nm of [^18^F]FMISO with that of known concentrations of unlabeled FMISO.

### Automated radiosynthesis of [^18^F]PM-PBB3 using [^18^F]5

After the [^18^F]F^−^ solution (7.4 ± 0.20 GBq, *n* = 11) was dried, a solution of epoxypropyl tosylate (**8**, 3.5 mg) in *o*-dichlorobenzene (0.15 mL) was added automatically to the reaction vial containing the dry [^18^F]F^−^ at 130 °C. The resulting [^18^F]**5** was distilled from the reaction vial under N_2_ flow at 30 mL/min and was transferred to another reaction vial containing precursor **7** (1 mg) and 1 mol/L NaOH (3.5 µL) in anhydrous DMF (0.25 mL) maintained at − 15 °C. After 2 min of trapping of [^18^F]**5**, the reaction mixture was heated at 130 °C for 20 min, and then was diluted with the preparative HPLC eluent (0.5 mL). The solution was transferred to the injector for preparative HPLC, as described in the general section. The HPLC conditions were as follows: Capcell Pak C_18_ column (5 μm, 10 mm i.d. × 250 mm length; Shiseido, Tokyo, Japan), a mixture of acetonitrile, water, and triethylamine (40:60:0.1, vol./vol./vol.) as the mobile phase, 5.0 mL/min flow rate, and UV detection at 365 nm. The retention time of [^18^F]PM-PBB3 was 14.9 min. The HPLC fraction of [^18^F]PM-PBB3 was collected in a flask containing polysorbate 80 (75 µL) in ethanol (0.3 mL) and ascorbic acid for injection (25 mg/0.1 mL water) was added before radiosynthesis. The solution was subsequently evaporated to dryness, and the residue was dissolved in physiological saline (3–10 mL). The solution of [^18^F]PM-PBB3 was passed through a Millex-GV filter to obtain [^18^F]PM-PBB3 as an injectable solution. The preparative HPLC and formulation were performed under UV-cut light (< 500 nm wavelength cutoff, ECOHiLUX HES-YF; Iris Oyama, Sendai, Japan) to prevent the photoisomerization of [^18^F]PM-PBB3, because [^18^F]PM-PBB3 underwent rapid photoisomerization upon exposure to fluorescent light (Kawamura et al. 2021).

The radiochemical purity of [^18^F]PM-PBB3 was determined by analytical HPLC under the following conditions: Atlantis T3 column (5 μm, 4.6 mm i.d. × 150 mm length; Waters), a mixture of acetonitrile and 50 mM ammonium acetate (pH 6.5) (40:60, vol./vol.) as the mobile phase, 1.0 mL/min flow rate, and UV detection at 365 nm. The retention time of [^18^F]PM-PBB3 was 12 min. The identity of [^18^F]PM-PBB3 was confirmed by co-injecting it with authentic unlabeled PM-PBB3. The molar activity of [^18^F]PM-PBB3 was measured using the same analytical HPLC system. The mass (µmol) of [^18^F]PM-PBB3 with a known radioactivity (GBq) was determined by comparing the UV absorbance at 365 nm of PM-PBB3 with that of known concentrations of unlabeled PM-PBB3. All of the above analytical processes were conducted in the absence of fluorescent light to prevent the photoisomerization of [^18^F]PM-PBB3.

## Results

### Automated radiosynthesis of [^18^F]FMISO using [^18^F]5

**Fig. 4:**
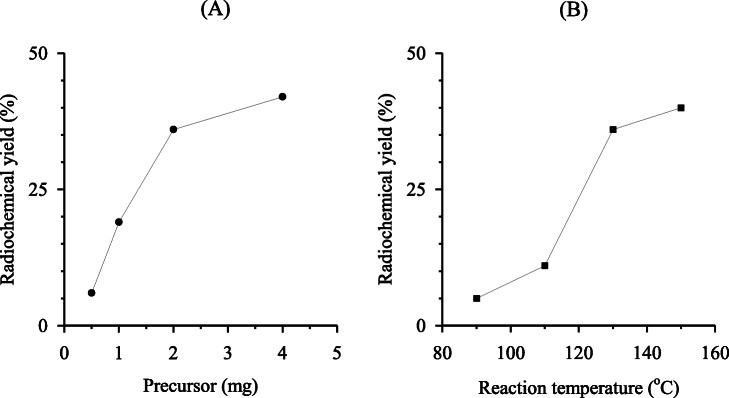
Radiochemical yields of [^18^F]FMISO via [^18^F]**5** in varied precursor dose (**A**) and reaction temperature (**B**). [^18^F]5 was distilled from the reaction vial and was transferred to the next reaction vial containing 6 (0.5, 1.0, 2.0 and 4.0 mg) and 1 mol/L NaOH (4.5, 9.0, 18 and 36 µL) in anhydrous DMF (0.25 mL) maintained at -15 °C. After 2 min of trapping of [^18^F]5, the reaction mixture was heated at 90, 110, 130, and 150 °C for 20 min to obtain [^18^F]FMISO

**Fig. 5:**
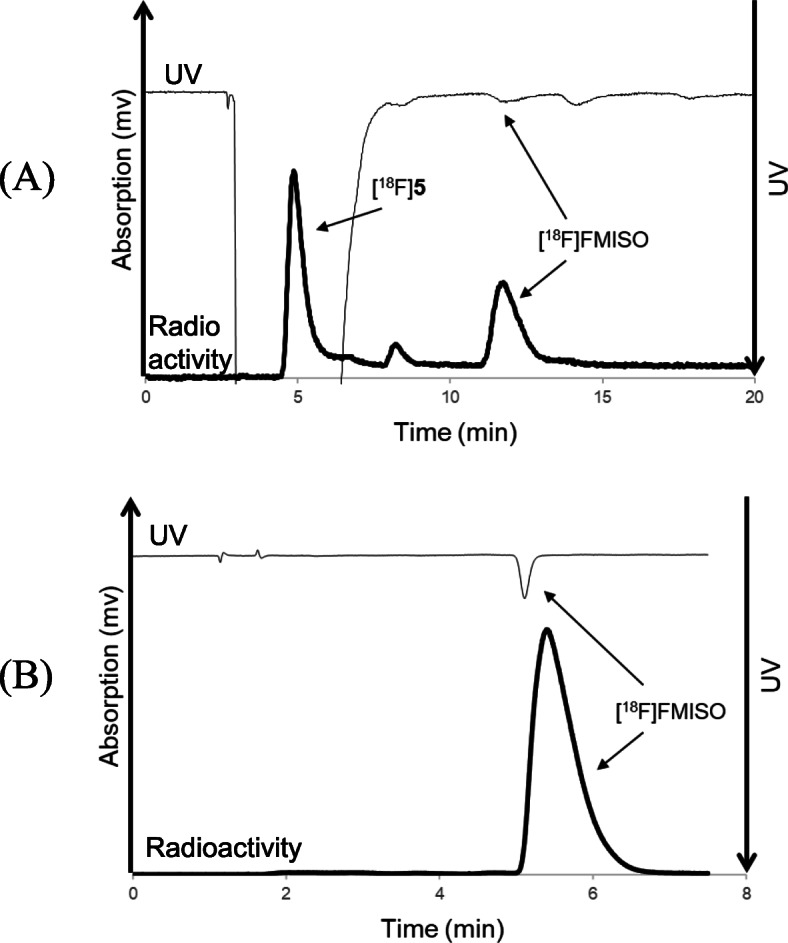
Preparative HPLC (**A**) and analytical HPLC (**B**) chromatograms of [^18^F]FMISO by ^18^F-fluoroalkylation using [^18^F]5. The preparative HPLC conditions were as follows: XBridge C_18_ column (5 μm, 10 mm i.d. × 250 mm length; Waters), a mixture of ethanol and water (2:98, vol./vol.) as the mobile phase, 5.0 mL/min flow rate, and UV detection at 325 nm. The retention time of [^18^F]FMISO was approximately 12 min (**A**). The analytical HPLC conditions were as follows: XBridge C_18_ column (5 μm, 4.6 mm i.d. × 150 mm length; Waters), a mixture of 90 % acetonitrile solution and 50 mM ammonium phosphate buffer (pH 9.3) (7:93, vol./vol.) as the mobile phase, 1.0 mL/min flow rate, and UV detection at 325 nm. The retention time of [^18^F]FMISO was 5.6 min (**B**)

We synthesized [^18^F]FMISO under various reaction conditions for the ^18^F-fluoroalkylation of 2-nitroimidazole precursor 6 and [^18^F]5 using an automated ^18^F-labeling synthesizer. With an increase in the amount of 6 from 0.5 to 4 mg, the radiochemical yield of [^18^F]FMISO gradually increased to 36 % from 0.5 to 2 mg, and marginally increased up to 42 % from 2 to 4 mg [Fig. 4(A)]. In addition, increasing the reaction temperature from 90 to 150 °C increased the radiochemical yield of [^18^F]FMISO by up to 40 % [Fig. 4(B)]. Furthermore, the radiochemical yield obtained by using sodium hydroxide (36 %) as a base for the reaction was slightly higher than that obtained by using sodium carbonate (22 %) or potassium hydroxide (24 %). From these results, we optimized the conditions for the radiosynthesis of [^18^F]FMISO using [^18^F]5 as follows: 2 mg of precursor 6, 18 µmol of sodium hydroxide as a base for the reaction, and a reaction temperature of 150 °C for 20 min. After completion of the reaction, preparative HPLC for the reaction mixture was performed to efficiently separate [^18^F]FMISO from the mixture, affording the radiochemically and chemically pure product as an injectable solution [Fig. 5(A)]. No significant UV peak corresponding to unreacted 6 and its decomposition components were observed in the analytical HPLC chromatogram of the final product solution [Fig. 5(B)].

Table 1 summarizes the results of the automated radiosynthesis of [^18^F]FMISO by ^18^F-fluoroalkylation using **6** and [^18^F]**5** for clinical applications. We successfully synthesized [^18^F]FMISO using [^18^F]**5**, with sufficient radioactivity (0.83 ± 0.2 GBq, *n* = 8) for clinical applications. The radiochemical yield of [^18^F]FMISO based on the cyclotron-produced [^18^F]F^−^ at the end of the synthesis (EOS) was 26 ± 7.5 % (*n* = 8). All the results of quality control for the [^18^F]FMISO injection complied with our in-house quality control and quality assurance specifications (Table 1).

**Table 1:** Radiosynthesis results of [^18^F]FMISO and [^18^F]PM-PBB3 by ^18^F-fluoroalkylation using precursor 6 and [^18^F]5

		[^18^F]FMISO	[^18^F]PM-PBB3
Cyclotron-produced [^18^F]F^−^ (GBq)		5.2 ± 0.20^b^	7.4 ± 0.20^d^
Radioactivity (GBq)^a^		0.83 ± 0.20^b^	0.79 ± 0.10^d^
Radiochemical yield (%)^a^		26 ± 7.5^b^*(40*^*c*^*)*	16 ± 3.2^d^*(25 ± 6.0*^*e*^*)*
Radiochemical purity (%)			
EOS		99 ± 0.50^b^	99 ± 0.50^d^
3 h after EOS		> 95^c^	> 95^d^
Synthesis time (min)		75 ± 4.0^b^	78 ± 4.0^d^
Molar activity (GBq/µmol)^a^		110 ± 20^c^	330 ± 140^d^

^a^At the end of synthesis (EOS)

^b^*n* = 8

^c^ the average result using ^18^F-fluorination in our routine radiosynthesis

^d^*n* = 11

^e^the result using ^18^F-fluorination (*n* = 53) (Kawamura et al. 2021)

### Automated radiosynthesis of [^18^F]PM-PBB3 using [^18^F]5

**Fig. 6:**
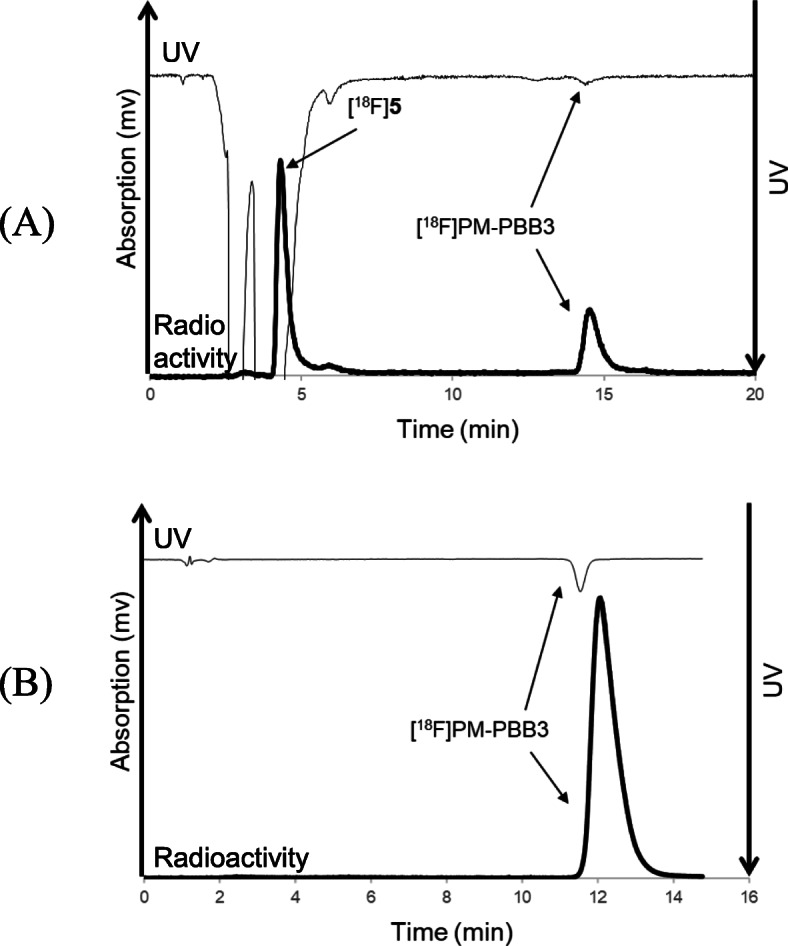
Preparative HPLC (**A**) and analytical HPLC (**B**) chromatograms of [^18^F]PM-PBB3 by ^18^F-fluoroalkylation using [^18^F]5. The preparative HPLC conditions were as follows: Capcell Pak C_18_ column (5 μm, 10 mm i.d. × 250 mm length; Shiseido), a mixture of acetonitrile, water and triethylamine (40:60:0.1, vol./vol./vol.) as the mobile phase, 5.0 mL/min flow rate, and UV detection at 365 nm. The retention time of [^18^F]PM-PBB3 was 14.9 min (**A**). The analytical HPLC conditions were as follows: Atlantis T3 column (5 μm, 4.6 mm i.d. × 150 mm length; Waters), a mixture of acetonitrile and 50 mM ammonium acetate (40:60, vol./vol.) as the mobile phase, 1.0 mL/min flow rate, and UV detection at 365 nm. The retention time of [^18^F]PM-PBB3 was 12 min (**B)**

We synthesized [^18^F]PM-PBB3 by the ^18^F-fluoroalkylation of precursor 7 and [^18^F]5 (Fig. 3), according to the reaction conditions previously determined for the reaction of a conventional phenol precursor with [^18^F]5 (Fujinaga et al. 2018). After the trapping of [^18^F]5 for 2 min, the ^18^F-fluoroalkylation of 7 and [^18^F]5 was performed at 130 °C for 20 min. The reaction mixture was then separated using preparative HPLC [Fig. 6(A)] to produce radiochemically and chemically pure [^18^F]PM-PBB3 as an injectable solution [Fig. 6(B)].

Table 1 summarizes the automated radiosynthesis results of [^18^F]PM-PBB3 by ^18^F-fluoroalkylation using precursor 7 and [^18^F]5 for clinical applications. We successfully synthesized [^18^F]PM-PBB3 using [^18^F]5, with sufficient radioactivity (0.79 ± 0.1 GBq, *n* = 11) for clinical applications. In addition, the radiochemical yield of [^18^F]PM-PBB3 based on the cyclotron-produced [^18^F]F^−^ at EOS was 16 ± 3.2 % (*n* = 11). All the results of quality control for the [^18^F]PM-PBB3 injection complied with our in-house quality control and quality assurance specifications (Table 1).

## Discussion

We successfully synthesized [^18^F]FMISO and [^18^F]PM-PBB3 by ^18^F-fluoroalkylation using [^18^F]5 with sufficient radioactivity for clinical applications. For [^18^F]FMISO, the radiochemical yield of the ^18^F-fluoroalkylation of 6 with [^18^F]5 was 26 ± 7.5 % (*n* = 8, Table 1), while the yield by the direct ^18^F-fluorination of 1 with [^18^F]F^−^ was approximately 40 % (from the average result in our routine radiosynthesis). For [^18^F]PM-PBB3, the radiochemical yield of the ^18^F-fluoroalkylation of 7 with [^18^F]5 was 16 ± 3.2 % (*n* = 11, Table 1), whereas the yield obtained by the direct ^18^F-fluorination of 2 with [^18^F]F^−^ was 25 ± 6.0 % (*n* = 53) (Kawamura et al. 2021). The reason for the difference in radiochemical yields between the two methods is the relatively lower reactivity of the corresponding precursors toward [^18^F]5 as a radiolabeling agent than toward [^18^F]F^−^. Moreover, the reactivity of [^18^F]5 seemed to be lower than that of conventional ^18^F-fluoroalkyl agents, such as [^18^F]fluoroethyl bromide and [^18^F]fluoroethyl iodide, toward the same phenol precursor. Recently, we found that the use of some Lewis acids could increase the reactivity of [^18^F]5 with aniline analogs (Fujinaga et al. 2019) and expect that the radiochemical yield of PET tracers containing the ^18^F-FHP moiety could be increased by ^18^F-fluoroalkylation using phenol or amine and [^18^F]5, catalyzed by a Lewis acid.

On the other hand, for direct ^18^F-fluorination, the tosylate precursors 1 and 2 should be synthesized with at least two steps from 6 to 7, respectively, and were limited to only the radiosynthesis of [^18^F]FMISO and [^18^F]PM-PBB3. For ^18^F-fluoroalkylation, imidazole precursor 6 and phenol precursor 7 are available and accessible. In particular, precursor 7 (PBB3) is an authentic unlabeled compound of [^11^ C]PBB3, which is a clinically used radiotracer for PET imaging of tauopathy in the human brain (Hashimoto et al. 2014, 2015; Maruyama et al. 2013). Moreover, 6 or 7 could be used to react with [^18^F]5 as well as other radiolabeling agents, such as [^11^ C]methyl iodide and ^18^F-fluoroalkyl agents, to produce diverse PET tracer candidates. A structure-activity relationship study is helpful for finding PET tracers with improved *in vitro* properties and *in vivo* behaviors by reacting the same precursor with diverse radiolabeling agents. This strategy has been applied to develop PET tracers in our group and to explore the best version from a series of candidates with the same chemical skeleton (Fujinaga et al. 2012; Zhang et al. 2003, 2004).

In this synthesis, the resulting [^18^F]**5** radiolabeling agent was purified by distillation from an ^18^F-fluorinated mixture of epoxytosylate 8 with [^18^F]F^−^, and was used for ^18^F-fluoroalkylation (Fujinaga et al. 2018). The distillation procedure removed all non-volatile impurities, such as metal ions from the cyclotron target, unreacted 8 and [^18^F]F^−^, and the phase transfer reagent Kryptofix 222 and K_2_CO_3_. Because of the utilization of purified [^18^F]5, only a small amount of precursor 6 (2 mg) or 7 (1 mg) was used for the ^18^F-fluoroalkylation, resulting in a clear ^18^F-fluoroalkylated reaction mixture. As shown in the respective HPLC separation charts for the reaction mixtures, in addition to the unreacted [^18^F]5, only the desired product corresponding to [^18^F]FMISO [Fig. 5(A)] or [^18^F]PM-PBB3 [Fig. 6(A)] peak was obtained from the reaction. Because of the large difference in the retention times of [^18^F]5 and [^18^F]FMISO or [^18^F]PM-PBB3, HPLC separation was easily conducted to obtain two radiochemically and chemically pure products [Fig. 5(B) and 6(B)]. Moreover, after ^18^F-fluoroalkylation, the reaction mixture did not require deprotection with acid, directly resulting in [^18^F]FMISO or [^18^F]PM-PBB3.

For direct ^18^F-fluorination, the tosylate precursor 1 or 2 is not stable in the presence of excess K_2_CO_3_ and Kryptofix 222 at high temperatures; therefore, a relatively large amount of 1 (5 mg) (Tang et al. 2005) or 2 (2 mg) (Kawamura et al. 2021) was required for the ^18^F-fluorination with [^18^F]F^−^ in order to produce sufficient radioactivity for clinical applications. The unreacted precursors and decomposed chemical components made the HPLC purification inconvenient (Supplemental information: Fig. S2 and S3). In the synthesis of [^18^F]FMISO by the ^18^F-fluorination using 1 and [^18^F]F^−^, after removal of the tosyl group in [^18^F]3 by treating the reaction mixture with HCl, *p*-toluenesulfonic acid (TsOH) was obtained. Only HPLC separation of the reaction mixture could not remove TsOH perfectly, and part of it would be left in the final product solution. Therefore, in our laboratory, after preparative HPLC for the reaction mixture of [^18^F]3 with HCl, the HPLC fraction was passed through a Sep-Pak cartridge (Cl^−^ form) to remove TsOH. In addition, [^18^F]FMISO was obtained as a chemically and radiochemically pure injectable solution.

## Conclusions

In this study, we successfully synthesized [^18^F]FMISO and [^18^F]PM-PBB3 by the ^18^F-fluoroalkylation using [^18^F]**5**, although the radiochemical yields of the ^18^F-fluoroalkylation using [^18^F]**5** were relatively lower than those of the corresponding direct ^18^F-fluorination using [^18^F]F^−^. Although the radiochemical yields were slightly lower for the synthesis route, the ^18^F-fluoroalkylations with [^18^F]**5** were cleaner and thus purification by HPLC alone yielded very pure products. Furthermore, we obtained relatively high chemical and radiochemical purity of [^18^F]FMISO or [^18^F]PM-PBB3 injection by radiosynthesis with the ^18^F-fluoroalkylation using [^18^F]**5**. Radiosynthesis using [^18^F]**5** is expected to be widely used to develop and produce useful PET tracers containing the ^18^F-FHP moiety.

## Supplementary Information

**Additional file 1: Figure S1.** The system diagram of automated multi-purpose synthesizer developed in-house (Fukumura et al. 2007). **Figure S2. **The preparative HPLC chromatogram of [^18^F]FMISO synthesized by ^18^F-fluorination using 1 and [^18^F]F^-^, followed by the removal of the protecting group in [^18^F]3. The HPLC conditions were as follows: XBridge C18 column (5 μm, 10 mm i.d. × 250 mm length; Waters), with a mixture of ethanol and water (2:98, vol./vol.) as the mobile phase, a flow rate of 5.0 mL/min, and UV detection at 325 nm. **Figure S3. **The preparative HPLC chromatograms of [^18^F]PM-PBB3 synthesized by ^18^F-fluorination using 2 and [^18^F]F^-^, followed by the removal of the protecting group in [^18^F]4. The HPLC conditions were as follows: Capcell Pak C18 column (5 μm, 10 mm i.d. × 250 mm length; Shiseido, Tokyo, Japan), the mixture of acetonitrile, water and triethylamine (40:60:0.1, v/v/v) as the mobile phase, 5.0 mL/min flow rate, and UV detection at 365 nm. 

## Data Availability

Data are provided in the article and supplementary information.

## Abbreviations

[^18^F]FMISO: [^18^F]Fluoromisonidazole
[^18^F]PM-PBB3: 1-[^18^F]Fluoro-3-((2-((1*E*,3*E*)-4-(6-(methylamino)pyridine-3-yl)buta-1,3-dien-1-yl)benzo[d]thiazol-6-yl)oxy)propan-2-ol
[^18^F]**5**: [^18^F]Epifluorohydrin
HPLC: High-performance liquid chromatography
PET: Positron emission tomography
^18^F-FHP: ^18^F-3-Fluoro-2-hydroxy)propyl
PBB3: 2-((1*E*,3*E*)-4-(6-(Methylamino)pyridin-3-yl)buta-1,3-dienyl)benzo[*d*]thiazol-6-ol
K_2_CO_3_: Potassium carbonate
Kryptofix 222: 4,7,13,16,21,24-Hexaoxa-1,10-diazabicyclo[8,8,8]hexacosane
CH_3_CN: Acetonitrile
NaOH: Sodium hydroxide
DMF: *N*,*N*-Dimethylformamide
TsOH: *p*-Toluenesulfonic acid
EOS: The end of synthesis

## References

[CR1] Arakawa R, Okumura M, Ito H, Seki C, Takahashi H, Takano H (2008). Quantitative analysis of norepinephrine transporter in the human brain using PET with (S,S)-^18^F-FMeNER-D_2_. J Nucl Med.

[CR2] Bruehlmeier M, Roelcke U, Schubiger PA, Ametamey SM (2004). Assessment of hypoxia and perfusion in human brain tumors using PET with ^18^F-fluoromisonidazole and ^15^O-H_2_O. J Nucl Med.

[CR3] Byun BH, Kim BI, Park SY, Ko IO, Lee KC, Kim KM (2017). Head-to-head comparison of ^11^ C-PiB and ^18^F-FC119S for Aβ imaging in healthy subjects, mild cognitive impairment patients, and Alzheimer’s disease patients. Med (Baltim).

[CR4] Chung SJ, Yoon HJ, Youn H, Kim MJ, Lee YS, Jeong JM (2018). ^18^F-FEDAC as a targeting agent for activated macrophages in DBA/1 mice with collagen-induced arthritis: comparison with ^18^F-FDG. J Nucl Med.

[CR5] Cole EL, Stewart MN, Littich R, Hoareau R, Scott PJH (2014). Radiosyntheses using fluorine-18: the art and science of late stage fluorination. Curr Top Med Chem.

[CR6] Deng X, Rong J, Wang L, Vasdev N, Zhang L, Josephson L (2019). Chemistry for positron emission tomography: recent advances in ^11^ C-, ^18^F-, ^13^ N-, and ^15^O-labeling reactions. Angew Chem Int Ed Engl.

[CR7] Eschmann SM, Paulsen F, Reimold M, Dittmann H, Welz S, Reischl G (2005). Prognostic impact of hypoxia imaging with ^18^F-misonidazole PET in non-small cell lung cancer and head and neck cancer before radiotherapy. J Nucl Med.

[CR8] Fujimura Y, Ikoma Y, Yasuno F, Suhara T, Ota M, Matsumoto R (2006). Quantitative analyses of ^18^F-FEDAA1106 binding to peripheral benzodiazepine receptors in living human brain. J Nucl Med.

[CR9] Fujinaga M, Yamasaki T, Yui J, Hatori A, Xie L, Kawamura K (2012). Synthesis and evaluation of novel radioligands for positron emission tomography imaging of metabotropic glutamate receptor subtype 1 (mGluR1) in rodent brain. J Med Chem.

[CR10] Fujinaga M, Ohkubo T, Yamasaki T, Zhang Y, Mori W, Hanyu M (2018). Automated synthesis of (rac)-, (R)-, and (S)-[^18^F]epifluorohydrin and their application for developing PET radiotracers containing a 3-[^18^F]fluoro-2-hydroxypropyl moiety. ChemMedChem.

[CR11] Fujinaga M, Ohkubo T, Kumata K, Nengaki N, Zhang MR (2019). Development of scandium-catalyzed N-[^18^F]fluoroalkylation of aryl and heteroaryl amines with [^18^F]epifluorohydrin. J Label Compd Radiopharm.

[CR12] Fukumura T, Suzuki H, Mukai K, Zhang MR, Yoshida Y, Nemoto K (2007). Development of versatile synthesis equipment for multiple production of PET radiopharmaceuticals. J Label Compd Radiopharm.

[CR13] Grierson JR, Link JM, Mathis CA, Rasey JS, Krohn KA (1989). A radiosynthesis of fluorine-18 fluoromisonidazole. J Nucl Med.

[CR14] Haneda E, Higuchi M, Maeda J, Inaji M, Okauchi T, Ando K (2007). In vivo mapping of substance P receptors in brains of laboratory animals by high-resolution imaging systems. Synapse.

[CR15] Harada R, Okamura N, Furumoto S, Furukawa K, Ishiki A, Tomita N (2016). ^18^F-THK5351: A novel PET radiotracer for imaging neurofibrillary pathology in Alzheimer disease. J Nucl Med.

[CR16] Harada R, Hayakawa Y, Ezura M, Lerdsirisuk P, Du Y, Ishikawa Y (2021). ^18^F-SMBT-1: a selective and reversible PET tracer for monoamine oxidase-B imaging. J Nucl Med.

[CR17] Hashimoto H, Kawamura K, Igarashi N, Takei M, Fujishiro T, Aihara Y (2014). Radiosynthesis, photoisomerization, biodistribution, and metabolite analysis of ^11^ C-PBB3 as a clinically useful PET probe for imaging of tau pathology. J Nucl Med.

[CR18] Hashimoto H, Kawamura K, Takei M, Igarashi N, Fujishiro T, Shiomi S (2015). Identification of a major radiometabolite of [^11^ C]PBB3. Nucl Med Biol.

[CR19] Hsu JL, Lin KJ, Hsiao IT, Huang KL, Liu CH, Wu HC (2020). The imaging features and clinical associations of a novel tau PET tracer-^18^F-APN1607 in Alzheimer disease. Clin Nucl Med.

[CR20] Iwata R, Pascali C, Bogni A, Furumoto S, Terasaki K, Yanai K (2002). [^18^F]Fluoromethyl triflate, a novel and reactive [^18^F]fluoromethylating agent: Preparation and application to the on-column preparation of [^18^F]fluorocholine. Appl Radiat Isot.

[CR21] Kämäräinen E-L, Kyllönen T, Nihtilä O, Björk H, Solin O (2004). Preparation of fluorine-18-labelled fluoromisonidazole using two different synthesis methods. J Label Comp Radiopharm.

[CR22] Kawamura K, Kumata K, Takei M, Furutsuka K, Hashimoto H, Ito T (2016). Efficient radiosynthesis and non-clinical safety tests of the TSPO radioprobe [^18^F]FEDAC: Prerequisites for clinical application. Nucl Med Biol.

[CR23] Kawamura K, Hashimoto H, Furutsuka K, Ohkubo T, Fujishiro T, Togashi T (2021). Radiosynthesis and quality control testing of the tau imaging positron emission tomography tracer [^18^F]PM-PBB3 for clinical applications. J Label Comp Radiopharm.

[CR24] Lee BS, Chu SY, Kwon HR, Park C, Sirion U, Brockschnieder D (2016). Synthesis and evaluation of 6-(3-[^18^F]fluoro-2-hydroxypropyl)-substituted 2-pyridylbenzothiophenes and 2-pyridylbenzothiazoles as potential PET tracers for imaging Aβ plaques. Bioorg Med Chem.

[CR25] Maruyama M, Shimada H, Suhara T, Shinotoh H, Ji B, Maeda J (2013). Imaging of tau pathology in a tauopathy mouse model and in Alzheimer patients compared to normal controls. Neuron.

[CR26] McCarthy TJ, Dence CS, Welch MJ (1993). Application of microwave heating to the synthesis of [^18^F]fluoromisonidazole. Appl Radiat Isot.

[CR27] Miller PW, Long NJ, Vilar R, Gee AD (2008). Synthesis of ^11^ C, ^18^F, ^15^O, and ^13^ N radiolabels for positron emission tomography. Angew Chem Int Ed Engl.

[CR28] Mori W, Takei M, Furutsuka K, Fujinaga M, Kumata K, Muto M (2017). Comparison between [^18^F]fluorination and [^18^F]fluoroethylation reactions for the synthesis of the PDE10A PET radiotracer [^18^F]MNI-659. Nucl Med Biol.

[CR29] Mori W, Yamasaki T, Fujinaga M, Ogawa M, Zhang Y, Hatori A (2019). Development of 2-(2-(3-(4-([^18^F]fluoromethoxy-d_2_)phenyl)-7-methyl-4-oxo-3,4-dihydroquinazolin-2-yl)ethyl)-4-isopropoxyisoindoline-1,3-dione for positron emission tomography imaging of phosphodiesterase 10A in the brain. J Med Chem.

[CR30] Oh SJ, Chi DY, Mosdzianowski C, Kim JY, Gil HS, Kang SH (2005). Fully automated synthesis of [^18^F]fluoromisonidazole using a conventional [^18^F]FDG module. Nucl Med Biol.

[CR31] Sasaki T, Ito H, Kimura Y, Arakawa R, Takano H, Seki C (2012). Quantification of dopamine transporter in human brain using PET with ^18^F-FE-PE2I. J Nucl Med.

[CR32] Su Y, Fu J, Yu J, Zhao Q, Guan, Zuo Y (2020). Tau PET imaging with [^18^F]PM-PBB3 in frontotemporal dementia with MAPT mutation. J Alzheimers Dis.

[CR33] Tagai K, Ono M, Kubota M, Kitamura S, Takahata K, Seki C (2021). High-contrast in vivo imaging of tau pathologies in Alzheimer’s and non-Alzheimer’s disease tauopathies. Neuron.

[CR34] Tago T, Furumoto S, Okamura N, Harada R, Adachi H, Ishikawa Y (2016). Structure-activity relationship of 2-arylquinolines as PET imaging tracers for tau pathology in Alzheimer disease. J Nucl Med.

[CR35] Tang G, Wang M, Tang X, Gan M, Luo L (2005). Fully automated one-pot synthesis of [^18^F]fluoromisonidazole. Nucl Med Biol.

[CR36] Weng CC, Hsiao IT, Yang QF, Yao CH, Tai CY (2020). Characterization of ^18^F-PM-PBB3 (^18^F-APN-1607) uptake in the rTg4510 mouse model of tauopathy. Molecules.

[CR37] Wilson AA, Dasilva JN, Houle S (1995). Synthesis of two radiofluorinated cocaine analogues using distilled 2-[^18^F]fluoroethyl bromide. Appl Radiat Isot.

[CR38] Xie L, Yui J, Hatori A, Yamasaki T, Kumata K, Wakizaka H (2012). Translocator protein (18 kDa), a potential molecular imaging biomarker for non-invasively distinguishing non-alcoholic fatty liver disease. J Hepatol.

[CR39] Yanamoto K, Kumata K, Yamasaki T, Odawara C, Kawamura K, Yui J (2009). [^18^F]FEAC and [^18^F]FEDAC: two novel positron emission tomography ligands for peripheral-type benzodiazepine receptor in the brain. Bioorg Med Chem Lett.

[CR40] Yang Y, Wang X, Yang H, Fu H, Zhang J, Zhang X (2016). Synthesis and monkey-PET study of (R)- and (S)-^18^F-labeled 2-arylbenzoheterocyclic derivatives as amyloid probes with distinctive in vivo kinetics. Mol Pharm.

[CR41] Yui J, Maeda J, Kumata K, Kawamura K, Yanamoto K, Hatori A (2010). ^18^F-FEAC and ^18^F-FEDAC: PET of the monkey brain and imaging of translocator protein (18 kDa) in the infarcted rat brain. J Nucl Med.

[CR42] Zhang MR, Tsuchiyama A, Haradahira T, Yoshida Y, Furutsuka K, Suzuki K (2002). Development of an automated system for synthesizing ^18^F-labeled compounds using [^18^F]fluoroethyl bromide as a synthetic precursor. Appl Radiat Isot.

[CR43] Zhang MR, Maeda J, Furutsuka K, Yoshida Y, Ogawa M, Suhara T (2003). [^18^F]FMDAA1106 and [^18^F]FEDAA1106: two positron-emitter labeled ligands for peripheral benzodiazepine receptor (PBR). Bioorg Med Chem Lett.

[CR44] Zhang MR, Maeda J, Ogawa M, Noguchi J, Ito T, Yoshida Y (2004). Development of a new radioligand, N-(5-fluoro-2-phenoxyphenyl)-N-(2-[^18^F]fluoroethyl-5-methoxybenzyl)acetamide, for PET imaging of peripheral benzodiazepine receptor in primate brain. J Med Chem.

[CR45] Zhang MR, Suzuki K (2007). [^18^F]Fluoroalkyl agents: synthesis, reactivity and application for development of PET ligands in molecular imaging. Curr Top Med Chem.

