# Supplementary material for: Automated radiosynthesis of two 18F-labeled tracers containing 3-fluoro-2-hydroxypropyl moiety, [18F]FMISO and [18F]PM-PBB3, via [18F]epifluorohydrin
Source: EJNMMI Radiopharm Chem. 2021 Jul 10;6:23. doi: 10.1186/s41181-021-00138-9 (PMC8272768; doi:10.1186/s41181-021-00138-9)
Supplement: Supplementary file 1 — Additional file 1: Figure S1. The system diagram of automated multi-purpose synthesizer developed in-house (Fukumura et al. 2007). Figure S2. The preparative HPLC chromatogram of [18F]FMISO synthesized by 18F-fluorination using 1 and [18F]F-, followed by the removal of the protecting group in [18F]3. The HPLC conditions were as follows: XBridge C18 column (5 μm, 10 mm i.d. × 250 mm length; Waters), with a mixture of ethanol and water (2:98, vol./vol.) as the mobile phase, a flow rate of 5.0 mL/min, and UV detection at 325 nm. Figure S3. The preparative HPLC chromatograms of [18F]PM-PBB3 synthesized by 18F-fluorination using 2 and [18F]F-, followed by the removal of the protecting group in [18F]4. The HPLC conditions were as follows: Capcell Pak C18 column (5 μm, 10 mm i.d. × 250 mm length; Shiseido, Tokyo, Japan), the mixture of acetonitrile, water and triethylamine (40:60:0.1, v/v/v) as the mobile phase, 5.0 mL/min flow rate, and UV detection at 365 nm. [file 41181_2021_138_MOESM1_ESM.docx]

**Supplementary information for:**

**Automated radiosynthesis of two ^18^F-labeled tracers containing 3-fluoro-2-hydroxypropyl moiety, [^18^F]FMISO and [^18^F]PM-PBB3, via [^18^F]epifluorohydrin**

Takayuki Ohkubo^1,2^, Yusuke Kurihara^1,2^, Masanao Ogawa^1,2^, Nobuki Nengaki^1,2^, Masayuki Fujinaga^1^, Wakana Mori^1^, Katsushi Kumata^1^, Masayuki Hanyu^1^, Kenji Furutsuka^2^, Hiroki Hashimoto^1^, Kazunori Kawamura^1*^, and Ming-Rong Zhang^1^

^1^Department of Advanced Nuclear Medicine Sciences, Institute for Quantum Medical Science, National Institutes for Quantum and Radiological Science and Technology, Chiba 263-8555, Japan

^2^SHI Accelerator Service Ltd., Tokyo 141-0032, Japan

*Correspondence: kawamura.kazunori@qst.go.jp

Department of Advanced Nuclear Medicine Sciences, Institute for Quantum Medical Science, National Institutes for Quantum and Radiological Science and Technology, 4-9-1 Anagawa, Inage-ku, Chiba 263-8555, Japan

S1. The system diagram of an automated multi-purpose synthesizer


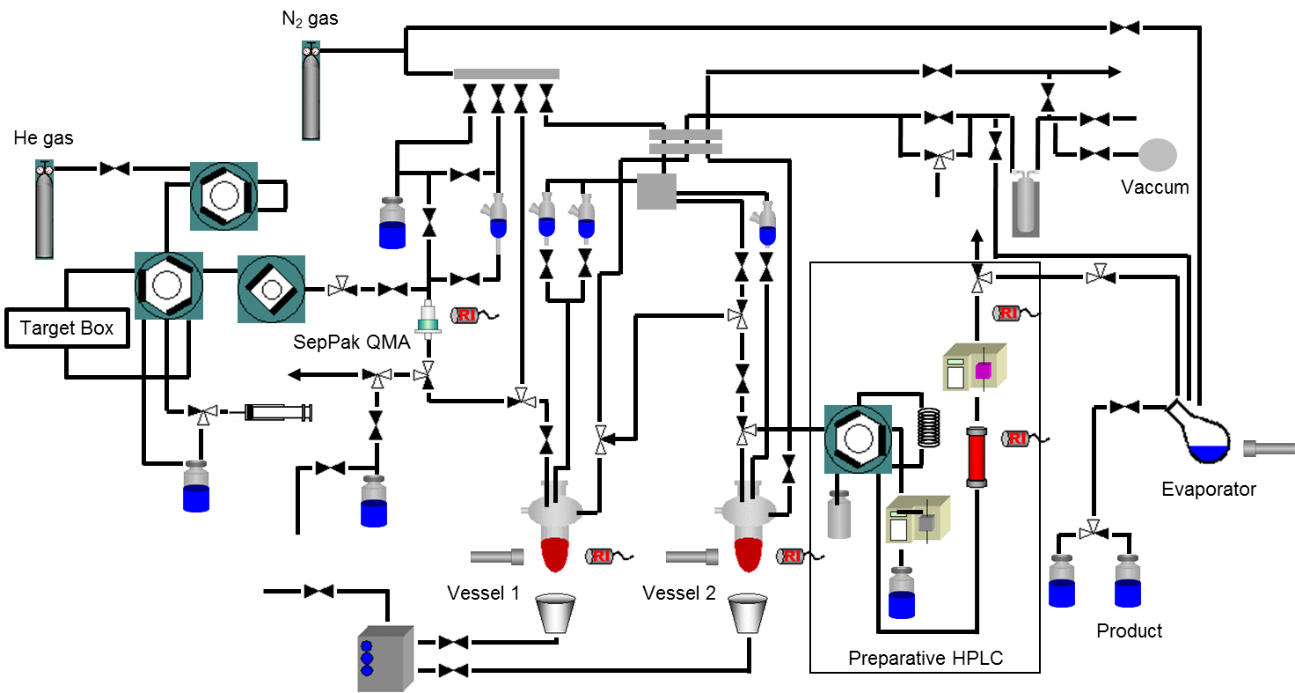


Fig. S1. The system diagram of automated multi-purpose synthesizer developed in-house (Fukumura et al. 2007)

S2. The preparative HPLC chromatogram of [^18^F]FMISO by ^18^F-fluorination


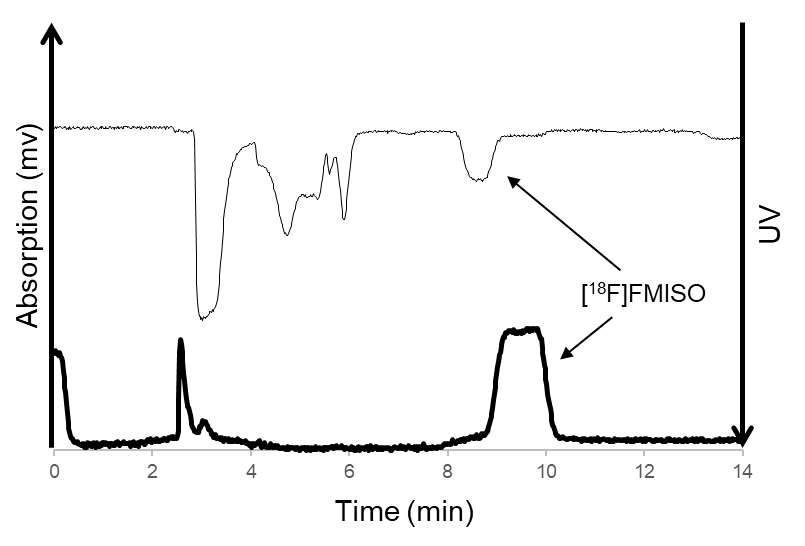


Radio

activity

UV

Fig. S2. The preparative HPLC chromatogram of [^18^F]FMISO synthesized by ^18^F-fluorination using **1** and [^18^F]F^-^, followed by the removal of the protecting group in [^18^F]**3**. The HPLC conditions were as follows: XBridge C18 column (5 μm, 10 mm i.d. × 250 mm length; Waters), with a mixture of ethanol and water (2:98, vol./vol.) as the mobile phase, a flow rate of 5.0 mL/min, and UV detection at 325 nm.

S3. The preparative HPLC chromatogram of [^18^F]PM-PBB3 by ^18^F-luorination


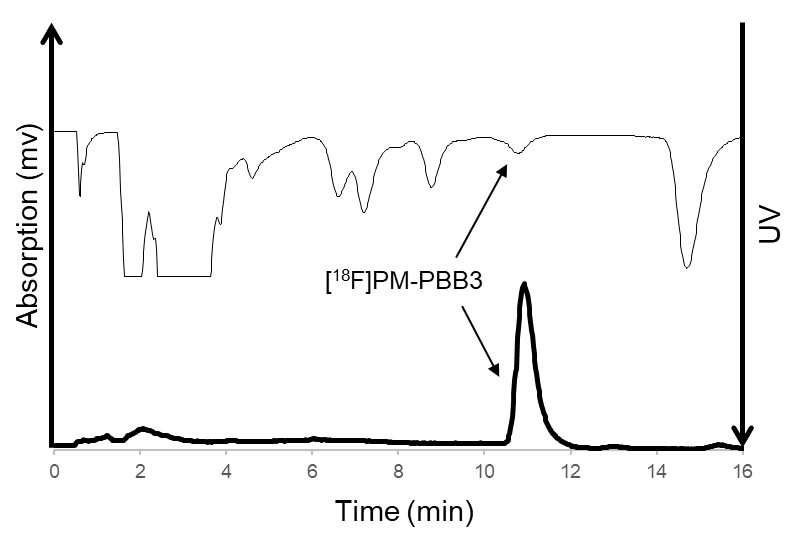


UV

Radioactivity

Fig. S3. The preparative HPLC chromatograms of [^18^F]PM-PBB3 synthesized by ^18^F-fluorination using **2** and [^18^F]F^-^, followed by the removal of the protecting group in [^18^F]**4**. The HPLC conditions were as follows: Capcell Pak C18 column (5 μm, 10 mm i.d. × 250 mm length; Shiseido, Tokyo, Japan), the mixture of acetonitrile, water and triethylamine (40:60:0.1, v/v/v) as the mobile phase, 5.0 mL/min flow rate, and UV detection at 365 nm.
